# Editorial: Old treatments new outcomes-augmentation treatments for depression

**DOI:** 10.3389/fpsyt.2024.1384641

**Published:** 2024-02-29

**Authors:** Nicolas A. Nuñez, Boney Joseph, Gustavo H. Vazquez

**Affiliations:** ^1^ Department of Psychiatry, University of Utah, Salt Lake City, UT, United States; ^2^ Department of Psychiatry & Psychology, Mayo Clinic, Rochester, MN, United States; ^3^ Department of Neurology, Mayo Clinic, Rochester, MN, United States; ^4^ Department of Psychiatry, Queen's University, Kingston, ON, Canada; ^5^ International Consortium for Mood & Psychotic Disorder Research, McLean Hospital, Belmont, MA, United States

**Keywords:** bipolar disorder, depression, augmentation strategies, systematic reviews, randomized controlled trials

## Introduction

Depression has become one of the most common and disabling mental disorders in the last years. Amongst adults, the literature has underscored a trend increase of depression (1) particularly with a worldwide prevalence increase of bipolar disorder (BD) reaching approximately 3% (2). Given that BD is a recurrent condition characterized by profound mood fluctuations spanning from mania/hypomania to severe depression, it is notable that the depressive phase of the illness contributes to a significant functional impairment and a diminished quality of life (3). In addition, established treatments have not modified drastically response and remission rates and in a few cases led to significant side effects that may contribute ultimately, to poor treatment adherence. Approximately one-third of patients do not respond and thus, may be considered treatment resistant (4). Different international guidelines have suggested multiple strategies such as a combination of different antidepressants or augmentation strategies with antipsychotics, mood stabilizers (lithium), or even different agents such as liothyronine and stimulants; however, the selection of these strategies still remains inconclusive with dearth of studies examining evidence base thoroughly (5). Augmentation strategies with compounds such as stimulants have been reported as early as the 1930s although concerns regarding substance abuse, risk for mood destabilization, and overall efficacy have been raised. Nevertheless, stimulant medications have been recognized in the literature as second and fourth-line therapy for patients specifically with BD. Specially of concern are how to select and personalize augmentation strategies in patients also with medical conditions. Additionally lifetime healthy behaviors such as exercise has shown its importance in reducing or alleviating depressive symptoms (6). Therefore, new ways of framing our clinical paradigms and integrating these strategies for depression and its management should be established. In this Research Topic we present selected studies that aim to highlight different augmentation strategies for patients with mood disorders, either with or without treatment resistance, with or without medical comorbidities or by improving residual symptoms such as daytime sleepiness and cognitive functions, all which could provide strategies to mitigate risk, personalize treatment and improve overall outcomes (see **Table 1** for an overview of included studies).

**Table 1:** Overview of edited primary studies.

Authors	Study type	Diagnosis	Country	Sample size/# studies	Participant age range or mean age	Intervention/Treatment	Behavior scales for depression	Primary outcome
Lu et al., 2023	RCT	Post-stroke depression	Chin	64	40-75	Electro-Acupuncture MRI-navigated rTMS treatment	HAMD-24	Clinical efficacy evaluated by change on HAMD-24
Lipschitz et al., 2023	RCT	Stable BD	USA	12	48.4	Modafini (100/200mg) in adjunctive to therapeutic dose of a mood stabilizer or placebo	HDRS	Effects of modafinil on neurocognitive function, daytime sleepiness and sleep quality in BD patients.
Yu et al., 2023	SR-MA	Adults with ESRD and depression	Chin	1059/ 22 studies	33.1-67.3	Exercis (aerobic, resistance, combined trainin)	BDI developed by Zun, HADS	Effects of exercise on depression in HD patients to assess the effectiveness of different exercise training parameters.
Qiu et al., 2023	MA	Adults with depression (MDD or TRD)	Chin	374/ 5 studies	35-49.4	Minocycline 200mg or placebo	HAMD-17 MADRS CGI BDI	Efficacy and safety of minocycline in depression

BD, bipolar disorder; BDI, Beck’s Depression Inventory; CGI, Clinical Global Impression; ESRD, end stage renal disease; HADS, Hospital Anxiety and Depression Scale; HAMD-17, 17-item Hamilton Depression Rating Scale; HAMD-24, Hamilton Depression Scale-24 items; HDRS, Hamilton Depression Rating Scale; MADRS, Montgomery-Asberg Depression Rating Scale; MA, meta-analysis; MDD, Major depressive disorder; RCT, randomized controlled trial; rTMS, repetitive transcranial magnetic stimulation; SDS, Zung Self-rating depression scale; SR, systematic review; TRD, treatment resistant depression; HD, hemodialysis.

## Randomized controlled trials

Lu and colleagues presented a protocol study in which 64 patients (age 40-75 years) with post stroke depression will be randomized to electro-acupuncture/MRI-navigated rTMS or only MRI-navigated rTMS for 12 to 20 sessions (4 weeks) assessing primarily changes on the Hamilton Depression Scale-24 item scores from baseline to 4 weeks. Additionally, the authors will measure behavioral scales assessing quality of life as well as acceptability and a cost-effectiveness analysis. Exploring the effectiveness of electroacupuncture, which has shown promise in treating depression, in conjunction with rTMS, could present a novel therapeutic strategy for individuals in this population (Lu et al.).

Lipschitz et al. aimed to better understand the effects of a stimulant like agent, modafinil on neurocognitive and sleep disturbances in patients with bipolar disorder. The authors randomized 12 stable BD patients to modafinil (100-200mg/day) or placebo adjunctive to mood stabilizers for a duration of 8 weeks. The authors underscored a cognitive benefit and overall improvement in daytime sleepiness in the experimental group. However, the authors did not find a significant difference in terms of sleep quality. In terms of emergent side effects, there were no significant differences with placebo, but the authors highlighted symptoms such as palpitations, itching, fatigue and decreased energy as new emergent side effects in the experimental group. This pilot study further enriches the existing literature by highlighting the potential enhancement and the role of stimulant-like agents in improving cognition, episodic memory, and working memory performance, thereby offering promise in alleviating cognitive challenges among patients with mood disorders (8).

## Systematic reviews

The first systematic review included in this series summarized evidence from 5 studies examining the utilization of minocycline—an antibiotic recognized for its anti-inflammatory, antioxidant, and neuroprotective attributes—in the treatment of psychiatric disorders. Qiu et al. suggested that minocycline in the included studies (minocycline, n=178; placebo, n=186) improved depressive symptoms and may augment response rates as well as it showed no statistically significant differences in all cause discontinuation rates when compared to placebo. This study provides more data of an affordable and readily available molecule which could potentially be effectively and safely utilized in TRD patients (9).

Considering the importance and the interplay between depressive symptoms and overall treatment outcomes in many medical conditions, Yu and colleagues conducted a systematic review in which they evaluated the effect of exercise on depressive symptoms in patients with comorbid end stage renal disease undergoing hemodialysis. By including 22 studies, they suggested that patients had better outcomes with intradialytic exercise and low depression following aerobic exercise; specially this was more notorious with activities with a duration greater than 1 hour. Moreover, those patients who engaged in exercise activities for more than 6 months had lower depression scores (Yu et al.).

The studies presented in this Research Topic provide an insight and overview of potential strategies for patients experiencing treatment resistance, residual symptoms with or without medical comorbidities. Moreover, the use of personalized interventions either by the use of these augmentation strategies, neuromodulation and lifestyle changes may provide an additional intervention to mitigate risk for relapse as well as to improve overall outcomes for individuals struggling with depression.

## Author contributions

NN: Conceptualization, Data curation, Formal analysis, Funding acquisition, Investigation, Methodology, Project administration, Resources, Software, Supervision, Validation, Visualization, Writing – original draft, Writing – review & editing. BJ: Conceptualization, Data curation, Formal analysis, Funding acquisition, Investigation, Methodology, Project administration, Resources, Software, Supervision, Validation, Visualization, Writing – original draft, Writing – review & editing. GV: Conceptualization, Data curation, Formal analysis, Funding acquisition, Investigation, Methodology, Project administration, Resources, Software, Supervision, Validation, Visualization, Writing – original draft, Writing – review & editing.
